# Characteristics and outcomes of elderly patients with Parkinson's disease hospitalized due to COVID‑19‑associated pneumonia

**DOI:** 10.3892/mi.2023.94

**Published:** 2023-07-04

**Authors:** Vasiliki Epameinondas Georgakopoulou, Aikaterini Gkoufa, Anastasia Bougea, Dimitrios Basoulis, Aristeidis Tsakanikas, Sotiria Makrodimitri, Georgios Karamanakos, Demetrios A. Spandidos, Efthalia Angelopoulou, Nikolaos V. Sipsas

**Affiliations:** 1 Department of Infectious Diseases and COVID-19 Unit, Laiko General Hospital, Medical School, National and Kapodistrian University of Athens, 11527 Athens, Greece; 2 Department of Pathophysiology, Laiko General Hospital, Medical School, National and Kapodistrian University of Athens, 11527 Athens, Greece; 3 1st Department of Neurology, Eginition Hospital, National and Kapodistrian University of Athens, 11528 Athens, Greece; 4 Laboratory of Clinical Virology, School of Medicine, University of Crete, 71003 Heraklion, Greece

**Keywords:** Parkinson's disease, pneumonia, coronavirus disease 2019, mortality, albumin

## Abstract

Patients with Parkinson's disease (PD) and coronavirus disease 2019 (COVID-19)-associated pneumonia present, according to the literature, high mortality rates due to the nature of the disease, advanced age, and underlying diseases. Most available studies, however, refer to the first waves of the pandemic. The aim of the present study was to investigate the clinical characteristics and outcomes of elderly patients (≥65 years old) with PD hospitalized with COVID-19-associated pneumonia during the period of prevalence of various severe acute respiratory syndrome coronavirus 2 (SARS-CoV-2) variants, as well as to determine possible prognostic factors for poor outcomes. During the period from February 15, 2021, to July 15, 2022, 1,144 elderly patients with COVID-19 pneumonia were hospitalized. Age, sex, Charlson comorbidity index, vaccination status against SARS-CoV-2, and admission laboratory parameters were recorded for all patients. A total of 36 (3.1%) patients with PD were hospitalized due to COVID-19-associated pneumonia (18 males, 50%). The mean age of the patients was 82.72±8.18 years. In total, 8 patients (22.2%) were hospitalized during the period of alpha variant predominance, 3 patients (8.3%) during the period of delta variant predominance, and 25 patients (69.4%) during the omicron variant predominance period. Of note, 16 patients (44.4%) were vaccinated with at least two doses. In addition, 17 (47.2%) patients succumbed to the disease. Between the patients who survived and those who succumbed, a statistically significant difference was only found in the mean value of albumin (37.48±6.02 vs. 31.97±5.34 g/l, P=0.019). In particular, as shown by receiver operating characteristic curve analysis, albumin exhibited a satisfactory predictive ability for mortality (area under the curve, 0.780; P=0.013) with an albumin value ≤37.7 g/l being able to predict mortality with 85.7% sensitivity and 54.8% specificity. Overall, the findings of the present study indicate that mortality among elderly patients with PD hospitalized with COVID-19-associated pneumonia was high in all phases of the pandemic. A low albumin value, not only as an indicator of the immune status, but also of the nutritional status, is a predictor of adverse outcomes.

## Introduction

Coronavirus disease 2019 (COVID-19), caused by the new severe acute respiratory syndrome coronavirus 2 (SARS-CoV-2), has had a marked impact on the worldwide civilization since December 2019. Globally, >760 million confirmed COVID-19 infections and 2.9 million related deaths have been reported as of May 7, 2023 (https://covid19.who.int/).

Apart from recurrent respiratory symptoms, neurologic manifestations occur in ~80% of hospitalized patients with COVID-19 at any point during the disease (1). Neurological entities, such as encephalitis, stroke, muscle injury, or anosmia have been linked to an increased risk of mortality in patients with COVID-19 (2,3). Furthermore, it is currently being debated whether pre-existing neurological diseases can become more severe following infection with COVID-19.

The COVID-19 pandemic has had an indirect and direct impact on individuals with Parkinson's disease (PD). Indirect impacts include a significant influence on healthcare provisions, such as a reduction in overall hospitalizations. Chronic diseases, on the other hand, require substantial delivery of care, including hospitalizations in situations of worsening of the main neurological disease or increased comorbidities. The direct effects of COVID-19 on PD were those that resulted in a substantial deterioration of motor and non-motor symptoms, which were attributable to both infection-related processes and poor dopaminergic pharmacokinetics (4-6).

PD has been shown to be associated with poor outcomes in patients with COVID-19-associated pneumonia (7). Considering the significant frequency of age-related comorbidities in patients with PD (8,9), it was determined that a putatively greater risk of mortality among patients with PD and COVID-19 is associated with frailty in older affected patients rather than with PD itself (10). Data on risk factors for mortality among patients with PD with COVID-19-associated pneumonia are limited. In addition, the studies that have been conducted to date aiming to evaluate the characteristics of patients with PD hospitalized for COVID-19-associated pneumonia and possible risk factors for adverse outcomes only refer to the first pandemic waves. Thus, the aim of the present study was to evaluate the clinical characteristics and outcomes of elderly patients with PD hospitalized due to COVID-19-assciated pneumonia over a long period of time during the pandemic, including the period of omicron variant predominance.

## Patients and methods

### Study design

The present study was a prospective study on elderly patients (≥65 years old) with PD and COVID-19-associated pneumonia hospitalized in the Department of Infectious Diseases and COVID-19 Unit of Laiko General Hospital (Athens, Greece) between February 15, 2021, and July 15, 2022. The research was conducted in line with the Declaration of Helsinki and obtained approval by the Institutional Review Board of Laiko General Hospital. Written informed was obtained from the patients for publication of their data. The following criteria for inclusion were applied: i) An age ≥65 years; ii) prior diagnosis of PD; iii) polymerase chain reaction confirmation of COVID-19 infection; iv) severe or critical disease according to the clinical spectrum of SARS-CoV-2 infection (https://www.covid19treatmentguidelines.nih.gov/).

### Data collection

Patient data regarding demographic characteristics, history, vaccination status against COVID-19, and the Charlson comorbidity index (CCI) were recorded. Hemoglobin and hematocrit levels, white blood cell, neutrophil, lymphocyte, platelet (PLT) and immature granulocyte counts, as well as the levels of C-reactive protein, serum albumin, lactate dehydrogenase, d-dimer, ferritin, liver and cholestatic enzyme were recorded upon admission.

### Statistical analysis

The Shapiro-Wilk test was used to assess the normal distribution of the parameters. Continuous parameters exhibiting a normal distribution are displayed as the mean (standard deviation), and those with a non-normal distribution are displayed as the median (range). The normally distributed continuous variables were compared using an unpaired t-test, and the comparison of non-normally distributed continuous variables was conducted using an unpaired non-parametric two-tailed Mann-Whitney U test. The examination of categorical variables was conducted using Fisher's exact or Chi-squared tests and these variables are presented as absolute numbers (frequency and percentage). The discriminative ability of significant parameters was assessed using receiver operating characteristic (ROC) curve analysis and the area under the ROC curve (AUC) was calculated. Statistical analyses were conducted using IBM SPSS-Statistics version 26.0 (IBM Corp.). Values of P<0.05 were considered to indicate statistically significant differences.

## Results

During the study period, 1,144 elderly patients with COVID-19-associated pneumonia were hospitalized in the Department of Infectious Diseases and COVID-19 Unit of Laiko General Hospital. A total of 36 (3.1%) patients with PD were hospitalized due to COVID-19-associated pneumonia (18 males, 50%).

The mean age of the patients was 82.72±8.18 years. In total, 8 (22.2%) patients were hospitalized during the period of alpha variant predominance, 3 (8.3%) patients during the period of delta variant predominance and 25 (69.4%) patients during the omicron variant predominance period. Of note, 16 patients (44.4%) were vaccinated with at least two doses. The most common comorbidity was dementia (15 patients, 41.7%), while 4 (11.1%) patients were nursing home residents. The demographics and clinical characteristics of the patients are presented in Table I.

Of the patients in the present study, 17 (47.2%) patients succumbed during their hospitalization. Between the patients who survived and those who succumbed, a statistically significant difference was only found in the mean value of albumin (37.48±6.02 vs. 31.97±5.34 g/l, P=0.019) (Tables II and III).

**Figure 1:**
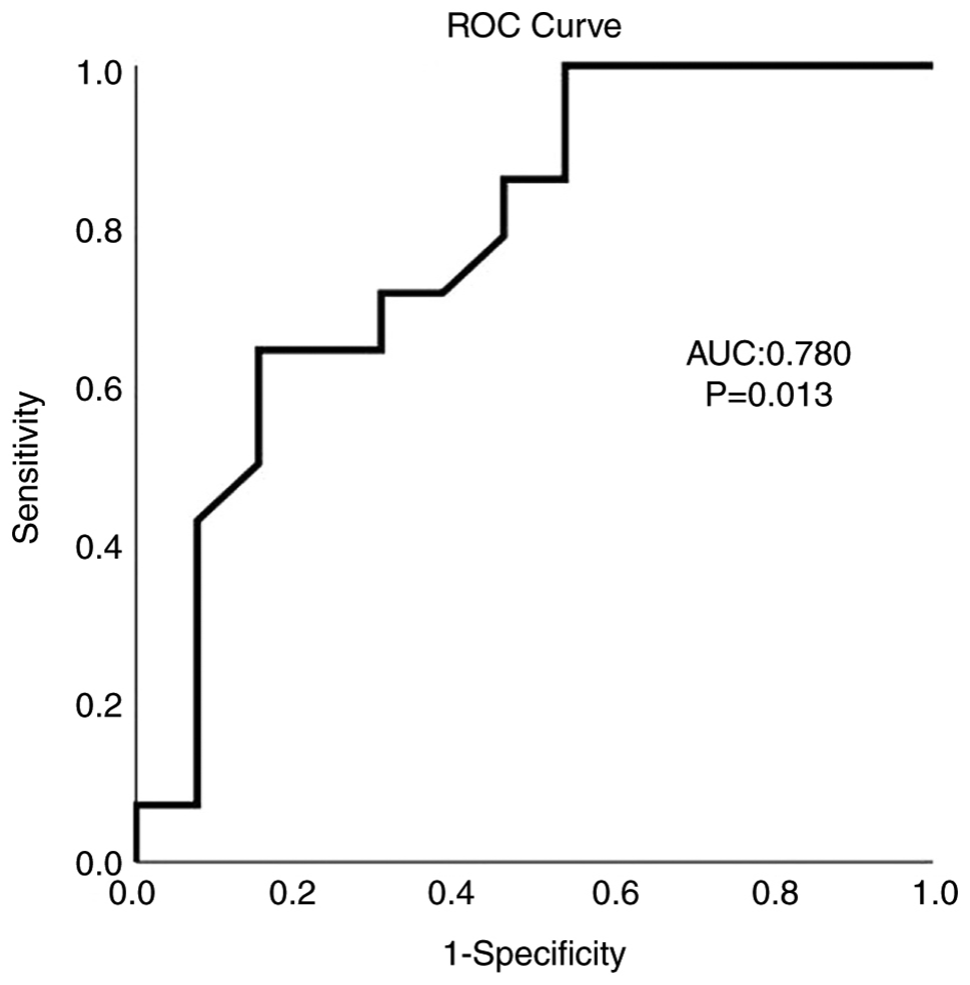
ROC curve analysis for albumin predicting mortality among patients with Parkinson's disease with COVID-19-associated pneumonia. AUC, 0.780; P=0.013. AUC, area under the curve; ROC, receiver operative characteristic.

As determined using ROC curve analysis, albumin exhibited a satisfactory predictive ability for mortality (AUC, 0.780; P=0.013) with an albumin value ≤37.7 g/l being able to predict mortality with 85.7% sensitivity and 54.8% specificity (Fig. 1).

## Discussion

As aforementioned, the available studies that have evaluated the mortality rates and the risk factors of adverse outcomes in patients PD are limited, mostly referring to the first periods of the pandemic. Scherbaum *et al* (11), in their cross-sectional study on the clinical profiles and mortality of inpatients with PD and COVID-19 in Germany, reported a mortality rate of 35.4%. Parihar *et al* (12) compared the outcomes of patients with PD with or without COVID-19 and found that patients with PD with SARS-CoV-2 infection had a mortality rate of 35.8%. Fathi *et al* (13), in their study, compared the prognosis of hospitalized patients with a known diagnosis of Alzheimer's or PD who had COVID-19 infection with that of other hospitalized patients with COVID-19 and reported a mortality rate of 35.1%. In addition, Salari *et al* (14), in their research exploring the mortality rates among patients with PD with COVID-19 in Iran, reported a mortality rate of 35.6% in these patients. In the present study, the mortality rate was 47.2%; however, the present study evaluated the clinical characteristics and outcomes of elderly patients with PD hospitalized with COVID-19-associated pneumonia over a long period of time during the pandemic.

Fortunately with the advancement of the vaccination campaign and the succession of SARS-CoV-2 mutations, the percentage of patients with COVID-19 with hospital admissions and COVID-19-associated mortality has changed significantly during the length of the pandemic, and the current trend in confirmed cases and deaths continues to decrease globally (https://covid19.who.int/). In the present study, no statistically significant differences were found in mortality rates between the periods of different variant predominance. This observation highlights the increased vulnerability of patients suffering from PD.

PD is one of the most prevalent age-related degenerative conditions, and it is frequently accompanied by comorbidities, notably cardiovascular disease (8); as a result, patients with PD are in the high-risk group for SARS CoV-2 infection-related mortality. Furthermore, there is an ‘indirect risk’ associated with the documented association between PD, age and cardiovascular comorbidities (15). Additionally, there is a direct risk of an unfavorable outcome following COVID-19 infection in patients with PD who already have respiratory impairment. Respiratory muscle weakness and aberrant posture, dictating respiratory muscle stiffness and insufficient respiratory excursions, contribute to ventilator failure in the advanced stage of PD (16,17). Pneumonia has been found to be the most prevalent reason for hospitalization and the leading cause of mortality among patients with PD, according to previous studies (16,18). In addition, 25-30% of patients with PD develop dementia, which is another risk factor for increased COVID-19-related mortality in these patients (19,20).

Several prognostic factors for mortality among hospitalized patients with PD with COVID-19 have been identified, such as age, race, an advanced PD stage, the reduction of PD medications and the presence of dementia (7,12-14). Serum albumin levels have been linked to poor outcomes in patients with COVID-19. As a typical nutritional indicator, low albumin levels in patients with COVID-19 may be a marker of excessive consumption due to tissue damage and hypermetabolism (21). A recent study demonstrated that higher serum albumin levels are significantly associated with improved cognitive function and play a protective role in motor impairment and PD-related mortality (22). To the best of our knowledge, the present study is the first to identify a laboratory parameter, serum albumin levels, as a prognostic factor for mortality among hospitalized patients with PD with COVID-19-assocaited pneumonia.

The present study has some limitations, however. Although it is a study referring to a long period of the pandemic, it is a single-center study with a relatively small number of patients. In addition, no medications regarding COVID-19 or other medications for the underlying disease were included in the analysis. Moreover, the stage of the disease and scales assessing the functional status of the patients were not included. Furthermore, viral variants were not identified individually for the participants. The assignment of variants was based on the predominant variant at the time the patient was diagnosed with the SARS-CoV2 infection.

In conclusion, the present study demonstrates that the mortality rate of elderly patients with PD hospitalized with COVID-19-associated pneumonia is high in all phases of the pandemic. A low albumin value, as an indicator of the immune status, as well as the nutritional status, is a predictor of adverse outcomes.

## Figures and Tables

**Table I:** Demographics and clinical characteristics of the study population.

Variable	Frequency
Sex, n (%)	
Female	18(50)
Male	18(50)
Variant, n (%)	
Alpha	8 (22.2)
Delta	3 (8.3)
Omicron	25 (69.4)
Comorbidities, n (%)	
Chronic lung disease (COPD/asthma/pulmonary fibrosis)	3 (8.3)
Cardiovascular disease (ischemic stroke, arrhythmia, coronary artery disease, myocardial infarction)	13 (36.1)
Arterial hypertension	9(25)
Diabetes mellitus	9(25)
Obesity	1 (2.8)
Chronic kidney disease	3 (8.3)
Dementia	15 (41.7)
Nursing home residency	4 (11.1)
Solid tumor malignancy	1 (2.8)
Hematological malignancy	2 (5.6)
Vaccination status, n (%)	
Fully vaccinated	16 (44.4)
Unvaccinated or partially vaccinated	20 (55.6)
In-hospital mortality, n (%)	
No	19 (52.8)
Yes	17 (47.2)
Age (years), mean (SD)	82.72 (8.18)
CCI, median (range)	5 (3-9)

CCI, Charlson comorbidity index; COPD, chronic obstructive pulmonary disease; SD, standard deviation.

**Table II:** Univariate analysis of continuous variables (outcome: Mortality).

Variable	Survivors	Non-survivors	P-value
Age (years), mean (SD)	81.05 (8.05)	84.59 (8.14)	0.200
Hb (g/dl), mean (SD)	12.27 (1.87)	11.23 (2.58)	0.173
Hct (%), mean (SD)	37.34 (5.49)	33.97 (7.62)	0.135
Neu (K/µl), mean (SD)	6.99 (4.18)	6.95 (4.49)	0.977
PLTs (K/µl), mean (SD)	234.73 (85.77)	216.52 (89.60)	0.539
LDH (U/l), mean (SD)	237.68 (116.92)	328.29 (157.07)	0.056
Albumin (g/l), mean (SD)	37.48 (6.02	31.97 (5.34)	**0.019**
CCI, median (range)	5 (3-9)	5 (3-9)	0.925
WBC (K/µl), median (range)	7.86 (2.1-21.45)	7.52 (3.06-19.37)	0.754
Lym (K/µl), median (range)	0.91 (0.26-3.21)	0.97 (0.23-1.53)	0.573
IGs (109/l), median (range)	0.05 (0.02-0.63)	0.05 (0.01-0.87)	0.639
Creatinine (mg/dl), median (range)	1.33 (0.48-7.4)	0.86 (0.44-5.36)	0.573
AST (U/l), median (range)	19.50 (11-42)	33 (13-67)	0.066
ALT (U/l), median (range)	11 (6-43)	10 (5-37)	0.639
ALP (U/l), median (range)	95.50 (30-175)	80 (41-220)	0.510
GGT (U/l), median (range)	19 (10-209)	36 (11-148)	0.232
CRP (mg/l), median (range)	27.59 (2.07-196.57)	46.25 (1.83-446)	0.639
Fer (ng/ml), median (range)	313 (32.6-1501)	537 (30.8-2340)	0.346
d-dimer (µg/ml), median (range)	1.48 (0.23-20)	2.45 (0.24-8.47)	0.292
FIB (mg/dl), median (range)	471.5 (90-666)	420 (255-957)	0.851
NLR, median (range)	7.75 (1-36)	9.30 (2-44)	0.802
PLR, median (range)	290.29 (81-816)	231.40 (76-1203)	0.999
CAR, median (range)	0.66 (0-9)	1.27 (2-21)	0.519

Values in bold font indicate statistically significant differences (P<0.05). ALP, alkaline phosphatase; ALT, alanine aminotransferase; AST, aspartate aminotransferase; CCI, Charlson comorbidity index; CRP, C-reactive protein; CAR, CRP-to-albumin ratio; Fer, ferritin; FIB, fibrinogen; GGT, gamma glutamyl-transferase; Hb, hemoglobin; Hct, hematocrit; IGs, immature granulocytes; LDH, lactate dehydrogenase; Lym, lymphocytes; NLR, neutrophil-to-lymphocyte ratio; Neu, neutrophils; PLTs, platelets; PLR, platelet-to-lymphocyte ratio; SD, standard deviation; WBC, white blood cell.

**Table III:** Univariate analysis of categorical variables (outcome: Mortality).

	No. of patients		
Variable	Survivors	Non-survivors	P-value
Sex			0.999
Female	10	8	
Male	9	9	
Vaccination status			0.756
Unvaccinated or partially vaccinated	10	10	
Fully vaccinated	9	7	
Variant			0.274
Alpha	6	2	
Delta	2	1	
Omicron	11	14	
Omicron variant			0.156
Yes	11	14	
No	8	3	
Chronic lung disease (COPD/asthma/pulmonary fibrosis)			0.087
Yes	3	0	
No	16	17	
Cardiovascular disease (ischemic stroke, arrhythmia, coronary artery disease, myocardial infarction)			0.502
Yes	8	5	
No	11	12	
Arterial hypertension			0.847
Yes	5	4	
No	14	13	
Diabetes mellitus			0.563
Yes	4	5	
No	15	12	
Obesity			0.337
Yes	1	0	
No	18	17	
Chronic kidney disease			0.481
Yes	1	2	
No	18	15	
Dementia			0.736
Yes	7	8	
No	12	9	
Nursing home residency			0.238
Yes	1	3	
No	18	14	
Solid tumor malignancy			0.337
Yes	1	0	
No	18	17	
Hematological malignancy			0.124
Yes	0	2	
No	19	15	

COPD, chronic obstructive pulmonary disease.

## Data Availability

The datasets used and/or analyzed during the current study are available from the corresponding author on reasonable request.
